# New Appliances and Things Medical

**Published:** 1910-10-29

**Authors:** 


### NEW APPLIANCES AND THINGS MEDICAL.

[We shall be glad to receive at our Office, 28 & 29 Southampton Street, Strand, London, W.C., from the manufacturers, specimens of all new preparations and appliances.]

### LAUNDRY MACHINERY.

(W. Summerscales and Sons, Ltd., Keighley.)

The Phœnix Foundry Works at Keighley are well known to institutional workers, for the firm of Summerscales has supplied many an institution with laundry, kitchen, and washing apparatus. The new catalogue issued by the firm is one of the fullest and most comprehensive we know, and gives details of many kinds of new patent apparatus which are worth trying by those who are not yet familiar with them. The price list, which is sent free on application to the firm, is admirably illustrated, and contains full descriptive letterpress, together with practical suggestions and hints which will be highly useful to those who have a mind to compare the apparatus with what is supplied by rival firms.

### DOULTON'S SANITARY APPLIANCES.

(Doulton and Co., Ltd., Lambeth, London, S.E.)

To hospital workers "Doultons" is sufficiently known to need no further introduction. The firm stands at the head of manufacturers of first-class sanitary ware, and nearly every hospital in the kingdom has experienced the advantage of having specimens of its manufacture fitted in the lavatories. We have always insisted upon the fact that it is false economy to fix inferior qualities of sanitary ware, and it cannot be too often explained that in the long run the best quality article proves the least costly. Messrs. Doultons' manufactures may be relied upon as being excellent in every way. The firm has a great reputation to keep up, and it is no exaggeration to say that it is adding to that reputation whenever it undertakes to fit out a new institution with sanitary appliances. We have just received a neatly printed and illustrated booklet describing Doultons' patent pneumatic water-waste preventing valve for w.c.s, and the fine model of water-closets—the "Astoria" pattern—which the firm supplies. Both are very suitable for institutional use. The valve, which is simple in design and excellent in construction, has been approved by the Metropolitan Water Board, and is silent, easily fixed, and so arranged that the supply of water can be modified as desired. Another feature of the firm's manufactures is the cast-iron porcelain enamelled bath: this is really a beautiful piece of work, enamelled, as all baths should be, both outside and inside, with an enamel which is of the most durable kind. Those who have not yet seen this bath, or any of Messrs. Doulton's specialties, would do well to inspect the large selection constantly on view at the showrooms at the Lambeth headquarters of the firm.

